# Magnesium Deficiency and Cardiometabolic Disease

**DOI:** 10.3390/nu15102355

**Published:** 2023-05-17

**Authors:** Remi Fritzen, Amy Davies, Miriam Veenhuizen, Matthew Campbell, Samantha J. Pitt, Ramzi A. Ajjan, Alan J. Stewart

**Affiliations:** 1 School of Medicine, University of St Andrews, St Andrews KY16 9TF, UK; rf79@st-andrews.ac.uk (R.F.);; 2 School of Nursing and Health Sciences, University of Sunderland, Sunderland SR1 3DS, UK; 3 Leeds Institute of Cardiovascular and Metabolic Medicine, University of Leeds, Leeds LS2 9JT, UK

**Keywords:** cardiovascular disease, magnesium, metal ion dyshomeostasis, nutrient deficiency, supplementation

## Abstract

Magnesium (Mg^2+^) has many physiological functions within the body. These include important roles in maintaining cardiovascular functioning, where it contributes to the regulation of cardiac excitation–contraction coupling, endothelial functioning and haemostasis. The haemostatic roles of Mg^2+^ impact upon both the protein and cellular arms of coagulation. In this review, we examine how Mg^2+^ homeostasis is maintained within the body and highlight the various molecular roles attributed to Mg^2+^ in the cardiovascular system. In addition, we describe how nutritional and/or disease-associated magnesium deficiency, seen in some metabolic conditions, has the potential to influence cardiac and vascular outcomes. Finally, we also examine the potential for magnesium supplements to be employed in the prevention and treatment of cardiovascular disorders and in the management of cardiometabolic health.

## 1. Introduction

Magnesium is an essential nutrient required by all forms of life [1]. In mammalian cells, Mg^2+^ is an abundant cation present at concentrations ranging from 5 to 20 mmol/L [2]. In the plasma, the magnesium concentration is a little lower at around 1 mmol/L. Many different reference values for serum magnesium have been proposed (as reviewed in [3]), which collectively suggest that the concentration ranges somewhere between ~0.6 and ~1.2 mmol/L in healthy humans. The Canadian Health Measure Survey Cycle 3, conducted in 2012–2013, measured serum magnesium in subjects aged 3–79 years. They reported that 9.5% to 16.6% of adults and 15.8% to 21.8% of adolescents (12–19 years) had serum magnesium concentrations < 0.75 mmol/L [4], which is a level currently accepted as an indication of magnesium deficiency. However, it has recently been suggested that this indicative value is likely to be too low and should be raised to <0.85, as values in this range are associated with increased health risks [3,5].

**Figure 1:**
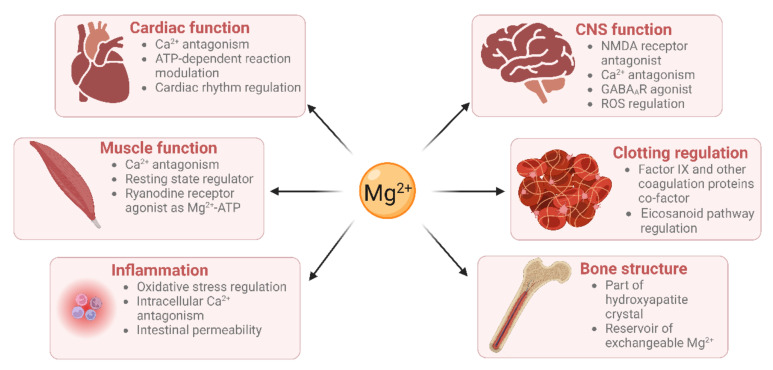
Magnesium has roles in many physiological processes (created using BioRender).

Mg^2+^ has many physiological functions, such as maintaining DNA and RNA stability, as well as regulating cellular proliferation, bone metabolism, and neuromuscular functioning [6,7], regulating inflammation [8], and maintaining haemostasis (Figure 1). Mg^2+^ is a co-factor for many enzymes [9]. These include protein kinases which are commonly utilised to regulate gene transcription in response to extracellular stimuli [10]. Mg^2+^ is also required for the structure and functioning of DNA and RNA polymerases [11,12]. These polymerases are not only involved in nucleic acid synthesis, but some are also involved in DNA repair and genome maintenance. Virtually all enzymes taking part in mismatch repair, nucleotide repair, and base excision repair use Mg^2+^ as a co-factor. Given that defects in genome maintenance pathways are considered a hallmark of many cancers, magnesium deficiency might contribute to oncogenesis [1]. Moreover, magnesium deficiency has been shown to be associated with diverse pathologies including (pre)diabetes mellitus, platelet hyper-reactivity, pre-eclampsia, acute myocardial infarction and even some therapies [13,14].

The scope of this narrative review extends to examine the importance of magnesium in cardiovascular and metabolic functioning and the influence of both dietary intake and supplementation on these systems. Here, we will consider how magnesium homeostasis is maintained and how an individuals’ magnesium status is assessed. We will also examine dietary magnesium deficiency in obesity and diabetes and review the roles magnesium plays in cardiovascular functioning and how pathologies caused by deficiencies may be alleviated through supplementation.

## 2. Magnesium Homeostasis

Magnesium homeostasis in the body largely depends on the collective actions of the intestine, skeleton and kidneys. The intestine is responsible for dietary uptake, the skeleton storage of ~50–60% of total Mg^2+^ in the form of hydroxyapatite, while the kidneys regulate its urinary excretion [1]. Magnesium can be found in all cells in the body [15,16], and it is particularly prevalent within mitochondria, the nucleus, and the endo/(sarco)-plasmic reticulum. The binding of Mg^2+^ by phospholipids, proteins, nucleic acids, chromatin, and nucleotides is thought to explain the presence of such high Mg^2+^ concentrations in these organelles [17].

Magnesium is naturally present in many foods; major sources include those of plant origin such as grains, vegetables, and pulses. In addition, magnesium is often added to food products and is available in various forms as a dietary supplement [18]. It has been known for some time that dietary magnesium intake is lower in Western populations due to industrial food processing that reduces the content of magnesium and other nutrients [19]. Reports also suggest that organic foods contain higher levels of magnesium than non-organic equivalents [20]. The United States Food and Nutrition Board recommends a daily Mg^2+^ intake of 420 mg for men and 320 mg for women [21]. When consumed as part of a meal, Mg^2+^ absorption is dependent on the overall composition of the meal. Some nutrients have an inhibitory effect (e.g., partly fermentable fibres or non-fermentable fibres), whilst others may increase Mg^2+^ absorption (e.g., low- or indigestible carbohydrates) [22].

Mg^2+^ absorption in the gut occurs via two separate pathways. Firstly, bulk absorption through the small intestine is thought to be regulated in a paracellular manner, since absorption correlates linearly with luminal Mg^2+^ concentrations [23,24]. Secondly, fine-tuning in the cecum and colon occurs transcellularly and involves the transient receptor potential (TRPM)-6 and 7 channels on the luminal enterocyte membrane for cell uptake [25] and the cyclin M4 transporter/exchanger on the basolateral membrane for Na^+^-dependent Mg^2+^ extrusion [26]. In contrast to other minerals, intestinal Mg^2+^ absorption is poorly regulated and depends mainly on intake [27,28]. Thus, overall Mg^2+^ maintenance and homeostasis are most likely regulated through excretion.

Sixty percent of the body’s total magnesium is stored in bones where it plays a structural role [29]. Two-thirds of this are stored within hydroxyapatite crystals. This portion is not readily available but is likely released following bone resorption [30]. Mg^2+^ binds at the surface of crystalline hydroxyapatite and aids in modulating crystal size and formation [31]. The quantity of magnesium present in the surface of the crystals is correlated with the plasma magnesium concentration, as demonstrated in studies with kidney disease patients [32]. This surface magnesium is a reservoir of readily exchangeable Mg^2+^ ions. Mg^2+^ deficiency affects the structure of bone, causing large hydroxyapatite crystals. It affects the cells involved in bone turnover, osteoblasts, and osteoclasts. Dietary magnesium intake has been linked to bone mineral density [33], and serum magnesium levels are strongly associated with an increased risk of fractures [33] and osteoporosis [34].

The excretion of Mg^2+^ is essentially regulated by filtration and reabsorption in the kidney [1]. Urinary Mg^2+^ excretion increases when magnesium intake is in excess, whereas the kidney conserves Mg^2+^ in the case of magnesium deprivation. Approximately one-tenth of total body magnesium is filtered by the kidney in a 24 h period [35]. A total of 10–15% of the filtered Mg^2+^ is reabsorbed in the proximal tubule by a passive process [36]. The majority of filtered Mg^2+^ (65%) is reabsorbed in the thick ascending loop of Henle [37], which is mediated by a paracellular mechanism dependent on the transepithelial potential generated by NaCl absorption. Thus, factors that impair NaCl reabsorption, such as diuretics and extracellular fluid volume expansion, increase Mg^2+^ excretion [38]. Around 10–15% of the filtered Mg is reabsorbed in the distal tubule [39]. The reabsorption occurs via an active transcellular mechanism and is regulated by divalent cation-sensing receptors.

The magnesium status of an individual is often determined by measuring the total serum or plasma magnesium concentration [40]. Plasma magnesium concentrations are closely related to bone metabolism, as there is continuous exchange between the skeleton and blood [41]. Since plasma/serum magnesium only represents 1% of the total magnesium in the body [42], it is possible that an individual can be in a Mg^2+^-depleted state but have plasma/serum values within the “normal” range. Consequently, the clinical impact of magnesium deficiency may be underestimated. In plasma, the concentration of free Mg^2+^ is reported to be ~0.6 mmol/L (~14 mg/L) [43], with ~30% complexed by proteins [44]. The major Mg^2+^-binding protein in plasma is serum albumin [45]. There are three structurally characterised Ca^2+^-binding sites on albumin [46], which are thought to also serve as Mg^2+^ sites. Mg^2+^ has also been shown to readily form complexes with globulin proteins in plasma [43].

## 3. Magnesium Concentration Measurement and Supplementation

Magnesium deficiency/insufficiency can present a diagnostic challenge, as patients may have a “normal” serum magnesium concentration but have relatively low levels of skeletal or cellular magnesium [33]. An indicator of intracellular magnesium status is the measurement of magnesium retention after acute magnesium loading. This is also known as the magnesium retention test. A magnesium deficiency is indicated if a patient has <80% excretion (over 24 h) of an infused magnesium load (2.4 mg/kg of lean body weight given over the initial 4 h) [47,48]. Additional tests for magnesium deficiency involve measuring the magnesium/creatinine ratio in spot urine or in 24 h urine collections [33]. It is also possible to directly measure magnesium in the urine; this can be used to gain insight into kidney functioning and magnesium wasting. A 24 h urinary magnesium level of > 24 mg is indicative of magnesium wasting [49].

Several studies have linked magnesium intake with the presence of certain cardiometabolic conditions [50,51,52,53,54,55,56,57]. However, it is important to consider the bioavailability of magnesium when analysing food intake, as it can vary greatly depending on the overall composition of the food as well as the quantity of magnesium present [58]. Low magnesium intake is particularly concerning in Western countries. For instance, about 75% of the Spanish population declared a dietary magnesium intake of less than 80% the recommended level [59,60]. Furthermore, a 2020 randomised controlled trial suggests the use of ionised Mg^2+^ as a preferred measurement for magnesium status rather than total magnesium, as the ionised form is the active form [61]. However, more studies are warranted to assess the usefulness of such measurements in a clinical setting.

Magnesium deficiency is commonly associated with other conditions including diabetes, obesity, infection, and malnutrition, while some commonly used therapies, such as proton pump inhibitors, can also cause significant magnesium deficiency [62]. Different magnesium salts have been used via multiple administration routes to treat some of the conditions linked to magnesium deficiency. However, single studies comparing the effect of different salts are rare. A recent randomised controlled trial by Schutten and colleagues compared the effect of magnesium citrate, oxide, and sulfate on arterial stiffness, measured as carotid-to-femoral pulse wave velocity, in 164 slightly obese or overweight but otherwise healthy patients over a period of 24 weeks [63]. Compared to placebo, they did not observe any significant effect with all three magnesium salts on carotid-to-femoral pulse wave velocity or blood pressure at 24 weeks compared with placebo. However, a subgroup analysis showed an amelioration in people with a higher baseline value, although the low number of participants in this subgroup did not allow a firm conclusion related to the different salts administered to be drawn. Effects on plasma magnesium were similar with each of the magnesium supplementation groups, but magnesium citrate led to a more pronounced increase in 24 h urinary excretion than magnesium oxide or magnesium sulfate. The study also recorded side effects related to the treatments and found that magnesium citrate and sulfate salts were more likely to lead to gastrointestinal complaints, which was not the case with magnesium oxide [63]. During supplementation, magnesium levels can take 20 to 40 weeks to reach a steady state; therefore, longer term studies with a higher number of participants are needed to fully establish the effects of magnesium supplementation [33]. Magnesium aspartate is also commonly given as a supplement to improve muscle weakness or cramps and displays high oral bioavailability and water solubility [64]. Other magnesium salts that are commonly used as supplements include magnesium orotate [65], magnesium pidolate [66], magnesium bisglycinate [67], magnesium malate [68] and magnesium acetyl taurate [68]. It is also important that plasma/serum magnesium levels are measured following supplementation to ensure compliance and avoidance of overdosing.

## 4. Magnesium Deficiency in Obesity and Diabetes

Diabetes mellitus is often associated with hypomagnesaemia. Patients with either type 1 diabetes mellitus (T1DM) or type 2 diabetes mellitus (T2DM) are more likely to have a low serum magnesium (<1.6 mg/dL or <0.66 mmol/L) than control patients without diabetes [69,70,71].

### 4.1. Type 1 Diabetes

T1DM is an autoimmune condition which leads to the destruction of β cells in the pancreas, resulting in a reduction in insulin production [71]. Exogenous insulin is required to treat people with T1DM to maintain normal serum glucose levels and magnesium deficiency (serum magnesium < 0.66 mmol/L has been reported in 4–38% of T1DM patients) [55]. When compared with age-matched controls, the mean plasma magnesium concentration was significantly lower in patients with T1DM [72]. The correlation between low magnesium and T1DM was particularly evident in female patients [72]. There is no evidence to suggest a direct mechanistic link between insulin and hypomagnesemia, although insulin might have an indirect role in the renal clearance of Mg^2+^.

Poorly controlled T1DM can lead to severe damage to the kidneys, eyes, and blood vessels [71]. Glycaemic control over the previous three months can be indicated by an HbA1c (glycated haemoglobin) test. HbA1c levels have been shown to negatively correlate with serum magnesium concentration in people with T1DM, suggesting that poor glycaemic control leads to hypomagnesaemia [71,72]. Other recent studies have found the incidence of hypomagnesaemia (<0.7 mmol/L) in patients with T1DM to be comparable to the general population [73]. Although the cohort was small, 207 participants were included, of which only nine had hypomagnesemia (4.3%). This finding was largely supported by a more recent study showing a hypomagnesaemia (<0.7 mmol/L) prevalence of 2.9% in people with T1DM [74]. However, markers of oxidative stress exhibited a negative correlation with magnesium levels, indicating that even a small reduction in magnesium level may have negative consequences. For reference, the prevalence of magnesium deficiency (<0.7 mmol/L) in the general population (i.e., without any known hypomagnesaemia risk factors) has been estimated to be around 2% [75]. A possible factor to consider is the success of treatment strategies for people with T1DM. Oost et al. noted that hypomagnesaemia seemed to be linked to glycaemia control only in patients who required high levels of insulin and displayed biochemical markers of insulin resistance, which is a population of patients with increased risk of diabetes complications [74].

### 4.2. Type 2 Diabetes and Obesity

T2DM is a condition characterised by a combination of defective insulin secretion and increased resistance to insulin by peripheral tissues [75,76]. Once T2DM has developed, individuals require treatment to reduce their serum blood glucose levels, including lifestyle advice and medications [77]. T2DM can develop more slowly than T1DM and can progress through a pre-diabetic phase. Metabolic syndrome refers to a collective of conditions including hypertension, insulin resistance, central obesity and atherogenic dyslipidaemia is a risk factor for T2DM [78,79]. Lifestyle factors such as diet and exercise are associated with metabolic syndrome as well as genetic and other environmental factors [80,81,82].

It has been suggested that hypomagnesaemia is caused by diabetes rather than contributing to T2DM onset, which is based on the findings of a cohort study reporting hypomagnesaemia (<0.7 mmol/L) being more common in patients with T2DM but not pre-diabetes [83]. However, other cohort studies challenge this. Indeed, the 2015 dose–response meta-analysis of prospective cohort studies published by Fang and colleagues found an inverse correlation between magnesium intake and T2DM [84]. The number of pooled participants totalled about 26,300 cases of T2DM with follow-ups ranging from 4 to 30 years, and the dietary magnesium intake was self-reported using a validated food frequency questionnaire. Moreover, in a 2017 metanalysis, which included 11 studies, Wu and colleagues found an inverse correlation between circulating magnesium concentration and T2DM as well as chronic heart disease and hypertension [85]. Finally, other cohort studies in both Western and non-Western populations have reported associations between magnesium and T2DM development [86,87].

Further evidence comes from intervention studies in individuals with poor metabolic health. A recent cost–benefit analysis study showed that 22.3% fewer men with pre-diabetes taking a magnesium supplement develop T2DM compared to placebo and supported such supplementation as a cost-effective preventative measure [88]. In addition, in a study of obese patients, serum magnesium levels increased by 13.2% and HbA1c decreased nine months post-bariatric surgery. This is likely to be due to a combination of weight loss, lifestyle changes and recommendation to take over the counter multi-vitamin tablets for four weeks [89].

It is also salient to highlight in this context genetic conditions in which individuals have an autosomal recessive genetic loss of function in TRPM-6 or 7, which contribute to magnesium homeostasis. These ion channels are found in the intestine and renal tubules and are important for magnesium exchange [90]. Importantly, people with these genetic conditions are more susceptible to hypomagnesaemia, and a possible association with T2DM has been documented, but large-scale studies are required to fully investigate this possibility [90].

Once T2DM has developed, evidence suggests that magnesium deficiency is still be treatable and may improve this condition. Magnesium deficiency has been shown to worsen the complications of the disease, whilst conversely, magnesium supplementation has been shown to protect against complications [91]. In addition, a meta-analysis revealed that magnesium supplementation on average seemed to improve levels of T2DM-associated biomarkers in T2DM patients. Although these trends did not reach significance, this might be attributed to the analysis including trials with a wide range of participant numbers and follow up times, with some not passing the 4-month threshold [92]. It is also notable that in a meta-analysis of 18 randomised controlled trials, T2DM individuals taking SGLT2 inhibitors (to reduce renal glucose absorption) had significantly higher serum magnesium compared to those taking the placebo. The authors concluded that further investigation is needed to understand clinical relevance, but it is tempting to speculate that SGLT2 inhibitors may confer part of their anti-T2DM effect in this way [93]. Current evidence supports the concept that magnesium supplementation may be a cost-effective way to decrease the risk of developing T2DM and minimise its harm post onset by improving risk biomarkers such as hypertension and glycaemic control.

## 5. Cardiovascular Roles of Magnesium

This section describes the physiological roles of magnesium in the cardiovascular system; in Section 6, we will address cardiovascular pathologies related to magnesium deficiency.

### 5.1. Cardiac Muscle Contraction

Mg^2+^ is an integral regulator of muscle contraction. Muscle contraction is a Ca^2+^-dependent process. Mg^2+^ can compete with Ca^2+^ for the binding sites on proteins involved in contraction, including the type-2 ryanodine receptor (RyR2), troponin C and myosin [94]. Before contraction, Mg^2+^ occupies all binding sites available in the myocyte, as its cytoplasmic concentration is 10,000-times higher than that of Ca^2+^. During excitation–contraction coupling, Ca^2+^ enters the cell, and Mg^2+^ is displaced from RyR2, allowing the channel to open and to release Ca^2+^ from the sarcoplasmic reticulum. The release of Ca^2+^ from intracellular stores can displace Mg^2+^ from the myosin head and troponin C, enabling contraction of the muscle [1].

Secondly, the effects of Mg^2+^ on the myocardium are protective against ischaemia and arrhythmia. The anti-ischaemic effects are the results of several factors. As with its vasodilatory properties, it prevents Ca^2+^ overload by competing for the same binding sites. It lowers the heart rate and contractility as well as catecholamine-induced oxygen demand. Moreover, it modulates ATP-dependent reactions and acts as an antioxidant to prevent long-term damage to the myocardium [95,96]. The anti-arrhythmic properties of Mg^2+^ are due to its modulation of voltage-gated Ca^2+^ channels and Na^+^ channels [97]. A protective effect on the myocardium has been shown in many *in vivo* studies, and magnesium is used as prophylaxis or treatment of myocardial complications after infarction or atrial fibrillation [95].

### 5.2. Vascular Functioning

The vascular system is the collective of vessels in the body which carry blood to and from tissues. Large blood vessels consist of three layers of tissue supported by the extracellular matrix. These are the adventitia where innervation is found, the media where smooth muscle cells are located, and the intima, which is lined by the endothelium and is in contact with the blood [98]. Studies support the concept that Mg^2+^ is important for several aspects of vascular functioning.

Vasodilation and vasoconstriction refer to the widening and narrowing of blood vessels, respectively. These processes allow blood flow to be matched to tissue demands. Mg^2+^ has been seen to improve blood flow in various vascular beds by dilating blood vessels [99,100,101]. Evidence suggests that this occurs in part because Mg^2+^ antagonises the transport of Ca^2+^ into contractile smooth muscle cells [102,103]. Multiple mechanisms may be involved [97]. These include the direct binding of Mg^2+^ to ion channels to block their activity [104] as well as the binding of Mg^2+^ to the plasma membrane, changing the surface charge and subsequently the opening of voltage-gated calcium channels [97]. In addition to direct actions on smooth muscle, studies also indicate that Mg^2+^ can modulate vascular dynamics by influencing signals from other tissues [98,105]. Firstly, Mg^2+^ has been documented to inhibit the release of noradrenaline, a vasoconstrictive neurohormone released in response to sympathetic stimuli, from nerve terminals [106]. Secondly, Mg^2+^ can act on the endothelium to alter its production of vasoactive compounds. Mg^2+^ has been shown to increase the production of the vasodilator’s nitric oxide and prostacyclin [107,108,109]. Notably, magnesium sulfate application has been linked to a reduced placental expression of endothelin 1, a potent vasoconstrictor [110].

There is evidence that Mg^2+^ is important *per se* for endothelial health and function. Culturing human endothelial cells *in vitro* in medium containing low magnesium leads to oxidative stress, inflammation, and the accumulation of lipids intracellularly [111,112]. Importantly, investigations indicate a vicious interplay to accelerate cell dysfunction [112]. For example, Mg^2+^-induced oxidative stress has been linked to the activation of NFkB [113]. NFkB is a transcription factor which induces the expression of pro-inflammatory cytokines as well as adhesion molecules which recruit monocytes and hence further aggravate inflammation and oxidative stress [113,114]. More recently, oxidative stress has been seen to promote lipid accumulation by increasing the activity of EDF-1, which is a transcriptional co-activator upstream of genes regulating lipid homeostasis [111]. In turn, intracellular lipids cause oxidative stress [115].

Another component of the vasculature for which Mg^2+^ appears important is the extracellular matrix (ECM). The make-up of the ECM depends on the blood vessel but is generally a complex composition and arrangement of elastic versus fibrous proteins. The ECM plays a pivotal role as a structural scaffold [116]. A matrix protein which contributes to structural integrity is hyaluronan, and Mg^2+^ is needed for the activity and correct folding of hyaluronan synthase [117]. The ECM additionally participates in a multitude of cell processes including vessel cell migration, adhesion, proliferation, differentiation, and survival [116]. Key proteins which allow the ECM to regulate vascular cell behaviour belong to the integrin family of transmembrane receptors. Integrins bind to ECM components and become activated, initiating intracellular signalling cascades [118]. Interestingly, integrins contain a metal ion-dependent adhesion site [119], and integrin–ligand interactions are dependent upon Mg^2+^ concentration [120,121,122]. For example, a major integrin in vascular tissue, α5β1, binds to the matrix protein fibronectin to promote vascular smooth muscle cell adhesion to the basal membrane. Studies suggest both that Mg^2+^ is needed to uncover α5β1 ligand binding sites [122] and that higher Mg^2+^ concentration leads to greater stability of α5β1–ligand interactions [121].

### 5.3. Haemostasis

Magnesium is involved in haemostasis as a co-factor for factor IX and membrane-bound coagulation proteins and as a regulator of the eicosanoid synthesis pathway, which produces inflammatory mediators including prostaglandins and thromboxane. Factor IX is part of the intrinsic pathway of the coagulation cascade, it activates factor X and is activated by activated factor VIII. The activation of factor IX is Ca^2+^-dependent [123]. Mutation of the factor IX gene is a hallmark of haemophilia B, a blood clotting disorder which is life threatening and shortens life expectancy [124]. Mg^2+^ has been shown to stabilise the native conformation of factor IX, and consequently to increase its activity [125]. Moreover, Mg^2+^ appears to be important for the early key stages of coagulation by enhancing the activity of the tissue factor-factor VIIa complex, which activates factor X [126].

Furthermore, during the initial stages of the coagulation process, when endothelial cell membranes are exposed to the blood stream, blood coagulation proteins reversibly interact with these membranes to trigger the coagulation cascade. Seven coagulation enzymes are bound to the cell surface through their γ-carboxyglutamate-rich (GLA) domains. GLA domain folding is dependent on both Ca^2+^ and Mg^2+^. The binding of these metal ions leads to the exposure of hydrophobic residues that ultimately help integration into the membrane bilayer. Under physiological conditions, the metal ions binding sites of GLA domains are occupied concurrently by Mg^2+^ and Ca^2+^, with two to three of the nine binding sites occupied by Mg^2+^ [127,128].

Finally, Mg^2+^ has been shown to inhibit the eicosanoid synthesis pathway in platelets. This pathway produces thromboxane which, once released, amplifies platelet aggregation. Magnesium sulfate is thought to modify platelet membrane fluidity, which in turn interferes with fibrinogen binding to the GPIIb/Iia complex and inhibits phosphoinositide breakdown and the formation of thromboxane [129]. Moreover, more recent research has shown that a similar inhibition occurs in macrophages using another magnesium salt, magnesium isoglycyrrhizinate. They showed that Mg^2+^ inhibits key enzymes involved in eicosanoid synthesis, which suggests that Mg^2+^ might have a direct inhibitory role on this pathway as well as through action on membrane fluidity [130].

## 6. Effects of Magnesium Deficiency on the Cardiovascular System

**Figure 2:**
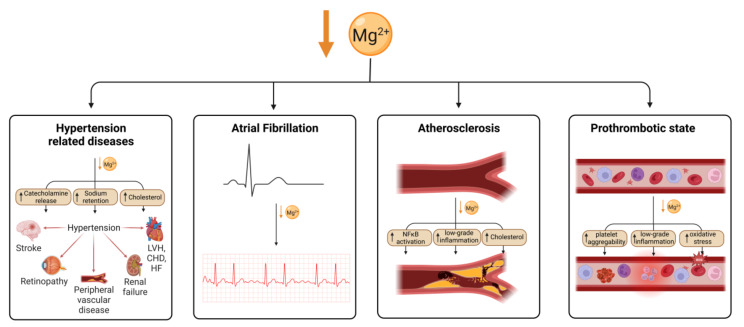
Magnesium deficiency leads to cardiovascular disease through multiple mechanisms. Mg deficiency leads to hypertension through an increase in catecholamine release, sodium retention and cholesterol, which is in turn a risk for several cardiovascular conditions. Arterial fibrillation has also been shown to be associated with Mg deficiency as well as atherosclerosis, which is thought to be caused by an increase in NFκB signalling, low-grade inflammation and cholesterol. Mg deficiency has also been linked to an increase in thrombotic risk. Created using BioRender.

Magnesium is an essential nutrient for cardiovascular health, acting to regulate vascular smooth muscle, cardiac conduction, vascular endothelial cell functioning, and thrombosis. Hypomagnesaemia and low dietary magnesium intake increase the likelihood of developing coronary artery disease (CAD) [131]. Hypomagnesaemia has been associated with hypertension, which can lead to congestive heart failure (CHF) or CAD (Figure 2). However, this could be confounded by diuretic medications to treat heart failure, which reduce serum magnesium levels in people with heart failure.

### 6.1. Hypertension

Hypertension is the term used when an individual has a systemic arterial blood pressure above 140/90 mmHg. Hypertension is a major risk factor for serious conditions including heart attack and stroke, peripheral arterial disease, and vascular dementia. It is therefore concerning that the percentage of adults diagnosed with hypertension rose from 549 million in 1975 to 1.13 billion in 2015 (WHO, 2023), representing a 3% rise from 12% to 15% of the world global population in 40 years. This increase is attributed to changing lifestyles with a widely established causative factor being the consumption of foods high in sodium. Less established is a link between Mg^2+^ intake and hypertension. Nevertheless, there is growing evidence indicating that appropriate Mg^2+^ consumption might help to recede hypertension prevalence.

There are several epidemiological studies which have examined the relationship between dietary magnesium and blood pressure. These include a large-scale cross-sectional study using data from the American National Health and Nutrition Examination Survey (NHANES). Three studies using different NHANES data sets consistently report inverse relationships between dietary Mg^2+^ and blood pressure and/or hypertension [50,51,52]. Importantly, corroborating trends have also been seen in cross-sectional analyses of European populations [53,54].

Further support for an inverse correlation is provided by longitudinal studies. This includes a 15-year follow-up of 4320 Americans [55], a mean nine-year follow-up of Mediterranean individuals [56], and follow-ups of 6104 subjects from China [57]. Further probing the relationship, the report by Dominguez et al. found that a dietary intake of magnesium below 200 mg/day is associated with a higher risk of hypertension [56], whilst Jiao et al. reported that individuals in the highest quantile of Mg^2+^ intake had a 20% lower risk of developing high blood pressure than those in the lowest quantile [57].

Notably, relationships between hypertension, Mg^2+^ and other dietary components have also been observed in epidemiological studies. Analysing NHANES data, it appeared that Ca^2+^ is protective against hypertension but only when women are taking recommended doses of Mg^2+^ and men are taking Mg^2+^ above recommended doses [50]. It has also been reported that Mg^2+^ significantly enhances the negative association between vitamin D and systolic blood pressure (SBP) [57]. These findings support that the magnitude of effect of Mg^2+^ intake on blood pressure (BP) will depend on the overall dietary profile of individuals.

As well as epidemiological studies, there is a sizeable repertoire of published Mg^2+^ supplement trials; however, currently available meta-analyses are inconsistent and may be limited because they are highly heterogenous [132]. Nevertheless, two large meta-analyses of randomised trials report that oral magnesium therapy significantly lowers blood pressure. In one study, the inclusion of 1173 individuals across 22 trials gave a median supplementation of 410 mg per day for 11 weeks and clinically relevant decreases of 3–4 mmHg and 2–3 mmHg in SBP and diastolic blood pressure (DBP) respectively [133]. Another study analysed 2028 participants across 34 trials and reported that a 368 mg/day dose for 12 weeks is sufficient to reduce SBP by 2 mmHg and DBP by 1.78 mmHg [134]. It is noteworthy that in this study, significant decreases were only found amongst individuals already taking antihypertensive or antidiabetic drugs. The authors suggest that this may be partly explained by the magnesium-lowering side effects of medications, which is consistent with the treated group beginning with serum Mg^2+^ below the clinical normal range. Furthermore, the previously reported study by Schutten and colleagues testing the effect of the administration of different Mg^2+^ salts for 24 weeks on blood pressure and arterial stiffness did not show any significant effect unless for a subgroup of participants with a higher baseline [63]. This lack of significant effect might be the result of a small sample size (164 participants) and/or a period of exposure too short. Overall, it seems that Mg^2+^ may help to reduce the incidence of hypertension. To better understand under which circumstances Mg^2+^ might be beneficial, future meta-analyses might be carried out to better assess heterogeneity across subgroups. It might also be interesting to explore supplement trials where Mg^2+^ is combined with other dietary compounds, such as vitamin D.

Pre-eclampsia is a pregnancy-specific hypertensive disorder that affects 2 to 8% of pregnancies and is responsible for the death of 63,000 women worldwide every year [135,136]. The cause of pre-eclampsia is not known, but hypomagnesaemia is known to be associated with the condition [137,138,139]. The usefulness of magnesium sulfate to treat pre-eclampsia was the subject of an international randomised controlled clinical trial, named MagPie, carried out on >10,000 women [140]. The participants were either given magnesium sulfate (*n* = 5071) or placebo (*n* = 5070). Although the development of side effects was greater (24%) in those given magnesium compared to placebo (5%), women allocated magnesium sulfate had a 58% lower risk of eclampsia (95% CI 40–71) than those allocated placebo (40, 0.8%, vs. 96, 1.9%; 11 fewer women with eclampsia per 1000 women). Mortality was also lower among women allocated magnesium sulfate (relative risk 0.55, 0.26–1.14) but there was no difference in risk of the baby dying. This suggests that magnesium supplementation can decrease the risk of eclampsia by over 50%.

### 6.2. Cardiac Functioning

In genome-wide association studies, high serum magnesium concentration has been associated with six single nucleotide polymorphisms. A 2018 mendelian randomisation study showed a causal relationship between high serum magnesium and lower risk of CAD [141]. This approach was also used in a more recent study to look at the association between high serum magnesium concentration, osteoporosis and cardiometabolic risks including T2DM, CAD, atrial fibrillation (AF) and heart failure. This second study found no association between cardiometabolic risks and serum magnesium level, whilst a strong causal relationship was found in the case of osteoporosis [142].

AF is a common and important risk factor of ischaemic stroke; this is due to clot formation in the atria of the heart which can embolise to the brain. People with AF are five-times more likely to have ischaemic strokes [143]. In the general population, low serum magnesium has been linked to an increased risk of AF. A long-term longitudinal study published in 2013 followed 3550 people for over 20 years with no prior history of cardiovascular disease. Over the period, 288 people developed AF, and the study shows a moderate association between low serum magnesium level and AF diagnosis [144].

Other cardiac conduction abnormalities such as prolongation of QT interval can lead to life-threatening cardiac arrhythmias. Hypomagnesaemia is associated with prolongation of QT interval [145]. In animal models of magnesium deficiency, QT prolongation was present, and cardiac myocytes were noted to have cardiac conduction abnormalities [144,146]. Inward-rectifying current and transient outward current were decreased in magnesium-deficient cardiomyocytes; transcription factors were also noted to be abnormal. Shimaoka et al. hypothesised that the above mechanism could be causing the increase in cardiac arrhythmias in patients with magnesium deficiency [147].

Hypomagnesaemia has been associated with heart failure, which is a condition where the heart fails to meet the circulatory demands of the body. A dose–response meta-analysis of prospective cohort studies found that an increase in dietary magnesium of 100 mg per day was associated with a 22% reduction in the risk of heart failure [84]. Magnesium is required for the normal production of all cells through its use in cellular respiration and ATP synthesis; therefore, magnesium deficiency could result in abnormal energy production in cardiac myocytes [33]. Due to the role of magnesium in excitation–contraction coupling, hypomagnesaemia could reduce the contractility of myocytes [33]. However, the number of pooled published dataset was limited to three, and they represented a total of 701 cases of documented heart failure. The same meta-analysis did not find a significant association between increased magnesium intake and CVD risk, whilst the highest magnesium dose intake (500 mg/day) category was associated with a 10% decrease in coronary heart disease risk.

The 4th International Study of Infarct Survival (ISIS-4) was a randomised factorial trial that assessed 58,050 patients entering 1086 hospitals up to 24 h (median 8 h) after the onset of suspected acute myocardial infarction (MI) [148]. Those with no clear contraindications to the study treatments (no cardiogenic shock or persistent severe hypotension) were randomised in a 2 × 2 × 2 factorial manner. Three treatment comparisons were assessed: (i) 1 month of oral captopril (6.25 mg initial dose titrated up to 50 mg twice daily) versus matching placebo; (ii) 1 month of oral controlled-release mononitrate (30 mg initial dose titrated up to 60 mg once daily) versus matching placebo; and (iii) 24 h of intravenous magnesium sulfate (8 mmol initial bolus followed by 72 mmol) versus open control. Whilst some benefits across the cohort were observed following captopril and mononitrate treatments, there was no significant reduction in 5-week mortality, either overall or in any subgroup examined, and further follow-up did not indicate any later survival advantage to those treated with magnesium.

In another magnesium supplementation study, the 2nd Leicester Intravenous Magnesium Intervention trial (LIMIT-2), the effect of an intravenous regimen of magnesium sulfate in 2316 patients with suspected acute myocardial infarction was assessed [149,150]. The study utilised a double-blind randomised protocol. Treatment was started with a loading injection, before any thrombolytic therapy, and continued with a maintenance infusion for a further 24 h. The cause-specific mortality of randomised patients was examined over 1.0–5.5 (mean 2.7) years of follow-up. It was found that the mortality rate from ischaemic heart disease reduced by 21% (95% CI 5–35%, *p* = 0.01) in magnesium-treated patients, and the all-cause mortality rate reduced by 16% (2–29%, *p* = 0.03). The study team attributed their positive results to the timing of the magnesium treatment. Such that for such protection to occur, magnesium must be raised by the time of reperfusion since the injury is immediate.

### 6.3. Vascular Disease

Hypomagnesaemia has been associated with an increase in cardiovascular mortality in patients with chronic kidney disease (CKD). One of the cardiovascular conditions associated with CKD is vascular calcification due to related hyperphosphatemia. Magnesium has an inhibitory effect on vascular calcification, and animal and human trials have shown a positive impact of supplementation [151]. Mechanistically, the preventative effect of magnesium may be due to the passive modulation of phosphate homeostasis and active regulation of vascular smooth muscle trans-differentiation [152].

Magnesium deficiency is associated with a chronic low-grade inflammation, including vascular inflammation. A deficiency of magnesium (<0.75 mmol/L) has been associated with an increased production of IL-1, IL-6, TNF-α, VCAM and PAI-1, which are proinflammatory molecules, and with a reduction in antioxidants such as glutathione peroxidase, superoxide dismutase, catalase, vitamin C, vitamin E and selenium [151]. Chronic low-level vascular inflammation has been implicated in endothelial dysfunction and vascular remodelling [153].

Greater intima-media thickness in the carotid arteries has been associated with hypomagnesaemia (<0.7 mmol/L) [33]. The measurement of intima media thickness in the carotid artery is a method to identify the presence of atherosclerotic plaque [154]; however, there are better methods available to detect atherosclerosis plaque, using B-mode ultrasound, multidetector CT (MDCT), or magnetic resonance imaging (MRI) [155]. A study published in 2010 used echography and 5-year follow-up to link serum magnesium at baseline and the difference in left ventricular mass. They found that lower serum Mg^2+^ was positively associated with higher left ventricular mass [156]. Another piece of evidence came from one cross-sectional 2022 study which used MRI to investigate a possible link between serum and the dietary intake of magnesium and subclinical markers of cardiovascular disease (left and right ventricular structure and function and carotid plaque and carotid wall thickness). Amongst 396 participants, 311 filled the magnesium intake survey. They did not find a correlation between all three variables, which was possibly due to the lack of correlation between serum magnesium and magnesium intake; however, serum magnesium was correlated with a higher risk of carotid plaque [155]. NFκB is one of the drivers of early atherosclerotic plaque formation, and magnesium deficiency has been shown to activate NFκB in endothelial cells *in vitro* [16,157]. Atherosclerosis in the carotid arteries is a risk factor for stroke, as emboli from these plaques can cause ischaemic stroke.

Peripheral arterial disease (PAD) is a manifestation of atherosclerosis and is diagnosed using symptoms or anatomical features as well as the measurement of the ankle-brachial pressure index (ABPI). The ABPI is the ratio of the SBP taken at the ankle to that in the arm. An ABPI of 1 is considered normal and an ABPI lower than 0.9 is sign of arterial/venous disease [158]. The atherosclerosis risk in communities (ARIC) is a prospective epidemiologic study undertaken in four US communities which ran from 1987 to 2013. It began with 15,792 participants aged 45 to 64 years old, who received five examinations throughout the length of the study. After exclusion, 13,826 participants were divided into five groups depending on their serum magnesium level as measured during the first visit. The investigators found a strong correlation between magnesium serum level and the prevalence of PAD as measured by an ABPI lower than 0.9 in subsequent visits or a hospital discharge with a diagnosis of PAD [159].

Mg^2+^ plays a role in platelet and endothelial function, including regulating the expression of signalling proteins. Hypomagnesaemia could therefore lead to prothrombotic and proatherogenic states [33]. Several mechanisms can lead to a prothrombotic state in magnesium deficiency, including low-grade vascular inflammation, increased platelet aggregation and oxidative stress in the vascular endothelium [160]. Although supplementation studies have looked at CVD risk association with hypomagnesaemia, studies are needed to assess a potential inverse relationship between thrombosis risk and magnesium intake. Because of the roles magnesium plays in coagulation, studies are warranted to explore a possible association between thrombotic risk, serum magnesium levels and/or magnesium intake.

Hypomagnesemia (<0.75 mmol/L) has been found by several independent studies to be a risk factor for cardiometabolic diseases, and many trials over the years have assessed the effect of magnesium supplementation on cardiometabolic risk [74,88,157]. Joris and colleagues published the results of a randomised controlled trial in 2016 in obese and overweight adults, looking at arterial stiffness, which is a marker of cardiovascular risk. Fifty-two male and female participants (age 62 ± 6 years) were randomised into two groups to receive either three daily doses of 117 mg of magnesium (total 350 mg per day) or placebo capsules for 24 weeks [161]. They measured arterial stiffness using carotid-to-femoral pulse wave velocity and found no significant improvement after 12 weeks, although they did not observe an increase in serum magnesium at that time. However, after 24 weeks, magnesium serum levels were increased, and they showed an improvement of the measure of arterial stiffness by 1.0 m/s (95% CI: 0.4, 1.6 m/s; *p* = 0.001). Unfortunately, they did not detect improvements in endothelial function and cardiometabolic risk markers in a subsequent report of the same study and concluded that some other factors might explain the previously observed improvement in arterial stiffness [162]. More recently, a 2020 small double-blind trial, which included 64 participants, studied the effect of magnesium sulfate supplementation daily for 3 months and showed improvement of some of the major risk factor for atherosclerosis, including HbA1c and oxidised low-density lipoprotein [163].

## 7. Conclusions and Future Perspectives

Magnesium plays a role in virtually all biological processes; it is a co-factor for many enzymes and competes with Ca^2+^ for binding to transporters. A deficiency of magnesium is associated with an increased risk of developing diseases such as cardiovascular and metabolic conditions. Consequently, studies looking at a wide range of common conditions (diabetes, hypertension, atrial fibrillation) suggest that supplementing the diet with Mg^2+^ may be beneficial. However, studies have often employed a limited period for magnesium supplementation and/or analysed small cohorts, making it difficult to draw definitive conclusions. Moreover, it seems that the choice of magnesium salt used might have an impact on the efficacy of such supplementation regimens. In addition, some disorders where magnesium deficiency may be of relevance and supplementation may be useful have not yet been fully examined: for instance, thrombotic conditions linked to aberrant haemostasis. Further well-designed and adequately powered studies are therefore urgently needed to fully understand the impact of magnesium deficiency and the potential benefits of magnesium supplementation on vascular pathology, cardiac function and the haemostatic process, particularly in higher-risk groups such as individuals with diabetes.

## Data Availability

Not applicable.

## Abbreviations

ABPI	ankle-brachial pressure index
AF	atrial fibrillation
ARIC	atherosclerosis risk in communities
BP	blood pressure
CAD	coronary artery disease
CI	confidence interval
CVD	cardiovascular disease
DBP	diastolic blood pressure
ECM	extracellular matrix
GABA_A_R	γ-aminobutyric acid A receptor
GLA	γ-carboxyglutamate-rich
HbA1c	glycated haemoglobin
HF	heart failure
IL-n	interleukin-n
LVH	left ventricular hypertrophy
NFκB	nuclear factor-kappa B
NMDA	N-methyl-D-aspartate
NHANES	American National Health and Nutrition Examination Survey
PAD	peripheral arterial disease
PAI-1	plasminogen activator inhibitor-1
ROS	reactive oxygen species
SBP	systolic blood pressure
T1DM	type-1 diabetes mellitus
T2DM	type-2 diabetes mellitus
TNF	tumour necrosis factor
TRPM	transient receptor potential channel
VCAM	vascular cell adhesion molecule
WHO	World Health Organisation
